# Supplementary material for: The interactome of intact mitochondria by cross-linking mass spectrometry provides evidence for coexisting respiratory supercomplexes
Source: Mol Cell Proteomics. 2017 Dec 8;17(2):216–32. doi: 10.1074/mcp.RA117.000470 (PMC5795388; doi:10.1074/mcp.RA117.000470)
Supplement: Supplemental Data [file supp_RA117.000470_133922_0_supp_23978_fzffwf.zip › spectra_annotation/mito_DR_spectra_annotation/231-1-3-1-16-1.pdf]

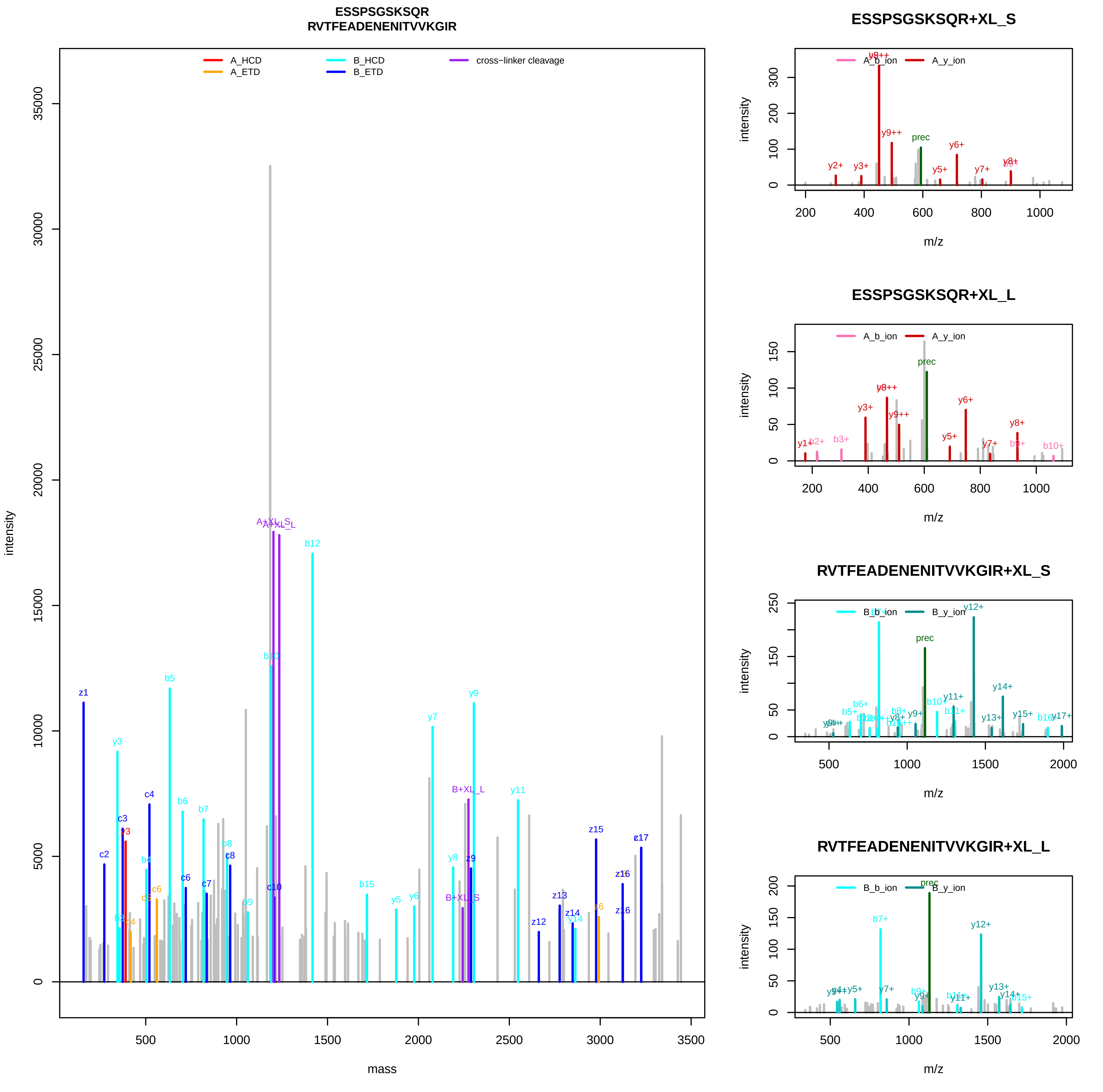
ESSPSGSKSQR
RVTFEADENENITVVKGIR
A_HCD
A_ETD
B_HCD
B_ETD
cross−linker cleavage
intensity
mass
z1
c2
y3
c3
y3
c4
b4
c5
c6
b5
b6
c6
b7
c7
b8
c8
b9
b10
A+XL_S
A+XL_L
c10
b12
b15
y5
y6
y7
y8
B+XL_L
B+XL_S
z9
y9
y11
z12
z13
z14
y14
z15
z6
z16
z17
ESSPSGSKSQR+XL_S
ESSPSGSKSQR+XL_L
RVTFEADENENITVVKGIR+XL_S
RVTFEADENENITVVKGIR+XL_L
A_b_ion
A_y_ion
B_b_ion
B_y_ion
y8++
y9++
prec
y2+
y3+
y5+
y6+
y7+
y8+
y1+
b2+
b3+
b9+
b10+
y12+
y14+
y15+
y13+
y11+
y9+
b5+
b6+
b8+
b10+
b16+
y17+
b7+
y4+
y5+
y7+
b9+
b11+
y13+
y14+
b15+
m/z
